# The Dividing Line

**Published:** 1887-08

**Authors:** Frank W. Low


### THE DIVIDING LINE.

Read at the Annual Meeting of the Eighth District Society, held in Buffalo, April 19 and 20, 1887.

BY DR. FRANK W. LOW.

"What an unaccountable thing, my friends," said Socrates on the morning before he drank the hemlock, "that seems to be which men call pleasure, and how wonderfully it is related to that which seems to be its contrary, called pain, in that they will not both be present to a man at the same time. Yet if a man pursues and overtakes the one, he is compelled to accept the other as if they were both united together to form one head." * * * "I suffered pain in my leg before from the chain, but now pleasure seems to have succeeded."

Pain is the shadow of pleasure, and never far from it. Indeed, by it we are sometimes made aware of the approach of pleasure, just as we are of the coming burst of sunshine by the scudding shadows that announce a breaking cloud. In a case of cholera morbus, as the pain of the agonizing cramp subsides, one realizes a sense of exquisite pleasure, like to nothing so much as an approaching burst of sunshine.

How can it be accounted for, that in one instant the same nerves can be made to thrill with sensations thus diametrically opposed to each other, kaleidoscoping the one into the other in such manner as to make it utterly impossible to draw the dividing line.

The apparent Siamese relationship of these twin sisters—pleasure and pain—is a problem that has challenged the thought of great minds in every age, from the days of Socrates to the present. A limited knowledge of metaphysics, coupled with considerable thought and the splendid opportunities for observation afforded at an operating chair, have led me to the following conclusions:

First—That all nerve impulse is transmitted like the forces of nature—light, heat, electricity and sound—in undulatory vibrations.

Second—That all nerve impulses transmitted from the terminals of the sensory nerve fibers are recognized at the ganglionic centers of sensation as either pleasurable or painful.

Third—That whether pleasurable or painful depends upon the intensity of impulse imparted to the nerve vibrations.

Upon these hypotheses I shall attempt to explain a few of the more remarkably peculiar manifestations of these sensations which have happened to come under my personal observation.

Independent of physical sensation, the mind is capable of experiencing emotional pleasure and pain. It is even capable of experiencing either of these, and at the same time its opposite physical sensation. Hope is a pleasurable emotion. Eminent surgeons agree that hope stimulates many a delicate young mother to bear the pains of parturition, who otherwise might have died of sheer exhaustion consequent upon prolonged labor pains.

Anger is a painful emotion, but there are women in whom, ordinarily, it is next to impossible to provoke the slightest sexual feeling, but who, under the excitement of intense anger, experience the liveliest erotic sensations. These metaphysical phenomena are in some degree above and beyond our explanation, but it is not improbable that while some of the centers of sensation in the brain are receiving impulses from terminal cells which are recognized as painful, the evolution of the mind should cause in other centers as pleasurable emotion. Leaving these out of the question then, let us return to the argument. Though recently vigorously opposed by another school of thinkers,* in support of my first proposition I must urge that it is the generally accepted theory that undulatory impulse is the law governing the forces of light, heat, electricity and sound. Recent writers even hold it to be the law of the forces of development. Now, as nerve force is demonstrated to be of the nature of electric force, it is fair to suppose it to be governed by the same law.

My second proposition, that all physical sensation is in some degree either pleasurable or painful, is, I believe, the universally accepted theory of metaphysics.

My third proposition, that whether pleasurable or painful depends upon the intensity of impulse transmitted, is a theory, so far as I know, never before advanced, to prove which shall be the labor of this essay.

It is the only theory, I think, that can be accepted in explanation of such phenomena as we are about to consider, while it is, I am equally sure, sufficient to explain any and all the phenomena of physical pleasure and pain. Why did Socrates suffer pain from the chain, and afterwards experience the sensation of pleasure? Why was the sensation following the cholera morbus cramp so pleasurable to me? Why is it that upon recovering consciousness after anæsthesia, occasionally a woman will accuse the operator with having taken improper liberties with her person? The anæsthesia cannot be accountable for this phenomena, else would it be presented when the operation was elsewhere than upon the teeth, and I half believe I might still further limit the occurrence to operations upon the teeth of the lower jaw. When not under the influence of an anæsthetic, in operations upon the inferior cuspid, bicuspid or molar teeth, why is it that if the interglobular spaces between dentine and en-

* Problem of Human Life. Substantial Philosophy etc., etc. A. Wilford Hall, Ph. D., LL. D.

amel are encroached upon the resultant paroxysm of pain is sometimes quickly followed by the liveliest sexual sensation?

That this is the experience of some women, I know; that it is the experience of many, I believe.

In the explanation of one of these phenomena we have furnished the key to the explanation of them all. According to the most generally accepted theory of atoms, not one in all the universe is ever still. The sources of heat—chemical, electric and solar—are sufficient, were there no other cause, to keep them constantly in motion. If one end of a bar of iron is thrust into a forge it soon becomes red hot. The atoms are vibrating with great intensity, and the bar expands to make more room for them. Yet could a particle of this metal, still glowing hot, be fixed beneath the glass of the most powerful microscope, we are told the vibration of the atoms would still be invisible. They do not change their relative positions in the bar, but each imparts its vibratory motion to its neighbor by contact.

Like to this bar of iron are our sensory nerve fibers. When the proper stimulant is brought in contact with a terminal atom, it is impelled to vibrate. If the stimulant be mild the vibration is slight, and the neighboring atom is gently jostled to vibrate in unison. Nerve force being more susceptible to impulse than the atoms of a bar of iron, the vibrations are more rapid, but by the same law of contact, and thus the centers of sensation, whether they be in ganglion or brain, are finally awakened. Was the stimulant delicate? The vibrations are gentle and the sensation is pleasurable. Had the stimulant been more intense the result would have been pain instead of pleasure.

A bar of iron is inorganic, and its atoms are incapable of tiring. If you continue to apply any degree of heat the vibrations will correspondingly continue, as is proven by the intensity of the brightness of the glowing rod. Nervous force pertains to organic matter, and its susceptibility may be exhausted. Although the degree of stimulation may remain unchanged, the vibratory impulse will sometimes gradually diminish, or suddenly cease altogether, from sheer exhaustion of the terminals. In one case we have pain gradually fading into pleasure before sensation ceases; in the other it suddenly ceases altogether. Death is sometimes caused by this sudden cessation—as, for instance, in fatal cases of angina pectoris.

There is no organic lesion of the heart, but simply paralyzation of the terminals of the pneumogastric nerve. They vibrate with such intensity that the acme of all pain is reached. Suddenly, from sheer exhaustion, the sensory terminal filaments cease to act, and the spur to the motor impulse being removed they refuse longer to cause tension and relaxation of the muscular heart fiber, and death ensues from its cessation.

It is a legitimate inquiry why pain inflicted upon a terminal cell of the inferior dental nerve should cause pleasurable sensation, especially in so remote a region as that of the sexual organs. A portion of the sensory impulse as it is impelled along the tract of the inferior dental nerve is switched off, as you might say, at the otic ganglion, and so sets to vibrating the sympathetic tract, and the phenomena of pleasurable instead of painful sensation in sympathy is accounted for, because it is a peculiarity of the sympathetic plexus* "that while it is endowed both with sensibility and the power of exciting motion, these properties are less active than in the cerebro-spinal system, and are exercised in an entirely different manner." An impulse, which in the dental nerve is sufficient to cause intense pain, becomes, by reflexion to the sympathetic, transmitted into a sensation of pleasure, while the slightest discomfort appreciable at the terminals of the latter is ofttimes magnified into the most painful neuralgic complications of the fifth pair. The eminent doctor Putzel says, in his recent work on functional nervous diseases,† of the causes of Trigeminal Neuralgia, "disorders of the genital organs and of the intestinal tract may also be enumerated." * * * "Sexual excesses are also injurious and very frequently act as the exciting cause."

Who will tell us why it is that we so often cry for joy, or that intense misery so frequently induces hysterical laughter? "The same nerves that thrill with pleasure thrill also with pain." "The same avenues that permit pleasure to enter the soul permit pain also to enter," and however assiduously these phenomena have been studied, it remains to science an open question still, where is the dividing line?

---

* Human Physiology, Dalton, page 499.

† Functional Diseases, Putzel, page 125.

